# Evolution of cefoperazone-sulbactam consumption in Romania between 2011 and 2024

**DOI:** 10.1007/s10096-026-05494-5

**Published:** 2026-05-05

**Authors:** Carmen-Cristina Vasile, Liviu-Iulian Rotaru, Roxana Ioana Șerban, Gabriel Adrian Popescu

**Affiliations:** 1 Carol Davila University of Medicine and Pharmacy, Bucharest, Romania; 2 National Institute of Infectious Diseases “Prof. Dr. Matei Balș”, Bucharest, Romania; 3 Department of Anatomy, Animal Physiology and Biophysics, Faculty of Biology, University of Bucharest, Bucharest, Romania; 4 National Centre for Communicable Diseases Prevention and Control, National Institute for Public Health, Bucharest, Romania; 5 Department of Molecular Biology, National Institute for Infectious Diseases “Prof. Dr. Matei Balș”, Dr. Calistrat Grozovici Street 1, Sector 2, Bucharest, 021105 Romania

**Keywords:** Cefoperazone-sulbactam, Fixed-dose combination, WHO AWaRe classification of antibiotics

## Abstract

**Purpose:**

The aim of this study is to describe the evolution of cefoperazone-sulbactam consumption in Romania between 2011 and 2024. The use of this fixed-dose combination is not recommended by the World Health Organisation as it may result in selection of antimicrobial resistance.

**Methods:**

This retrospective study analyzed the data on antibiotic consumption collected by the National Center for Surveillance and Control of Communicable Diseases which is based on data provided by the National Health Insurance Fund and the IQVIA–Multinational Integrated Data Analysis System. Antibiotic sales were converted into defined daily doses (DDDs) per 1,000 inhabitants per day.

**Results:**

The consumption of cefoperazone-sulbactam in Romania between 2011 and 2024 ranged from 0.016 to 0.021 DDD per 1000 inhabitants per day, with an average of 0.019 DDD per 1000 inhabitants per day. When adjusted for population, the RO1 macroregion registered the highest average consumption (0.033 DDDs per 1000 inhabitants per day). One-way ANOVA on DDD per 1,000 inhabitants per day data suggested that there were no statistically significant differences between macroregions (F = 2.78, p = 0.054). Although differences are observed in the mean values, they can be explained by counties with abnormally high consumption.

**Conclusion:**

Educational and training activities focused on the outlier counties are necessary to raise awareness about the risk of increased selection of antimicrobial resistance. In line with the rejection of the reclassification proposal of cefoperazone-sulbactam in the updated 2025 WHO AWaRe classification of antibiotics, new government policies to ensure the discontinuation of cefoperazone-sulbactam use in Romania are needed.

## Introduction

According to the Point Prevalence Survey (PPS) of Healthcare-associated infections (HAIs) and antimicrobial use in European acute care hospitals in 2022 and 2023, Romania reported the highest composite index of antimicrobial resistance (68.7%), with more than 80% of *Acinetobacter baumannii* isolates from HAIs being resistant to carbapenems [1]. According to the same study, 8.6% of total HAIs were caused by *Acinetobacter* spp. compared with the EU/EEA mean of 3.2%. More than 75% of invasive isolates of *Acinetobacter baumannii* reported by Romania between 2014 and 2024 were characterized by combined resistance to carbapenemes, fluoroquinolones and aminoglycosides [2].

Sulbactam, an irreversible mechanism-based beta-lactamase inhibitor, possesses intrinsic activity against *Acinetobacter* spp. and has been used in high doses to treat multidrug-resistant *Acinetobacter* spp. infections [3]. It is available as an FDC with ampicillin, cefoperazone or durlobactam. Sulbactam-durlobactam in combination with a carbapenem is the recommended regimen by the Infectious Diseases Society of America (IDSA) for the treatment of carbapenem-resistant *Acinetobacter baumannii* (CRAB) [4], but it has not yet been approved by the European Medicines Agency (EMA). The alternative regimen which is available in Europe is high-dose ampicillin-sulbactam in combination with at least one other agent (i.e., polymyxin B, tigecycline or cefiderocol). The recommended total daily dose of the sulbactam component is 9 g [5].

Administering suboptimal doses of sulbactam (4 g) as per the cefoperazone-sulbactam drug indications instead of the IDSA recommended dose of 9 g can lead to the development of *Acinetobacter* spp. resistance [6] via antibiotic selection pressure in the already very limited landscape of treatment for CRAB.

The World Health Organization introduced the AWaRe classification of antibiotics in 2017 to support antimicrobial stewardship programmes and reduce antimicrobial resistance. Antibiotics are classified as *access*,* watch*,* reserve* and *not recommended* for use on the basis of their clinical importance and the risk of their use promoting resistance [7]. According to the AWaRe classification, the use of the fixed-dose combination (FDC) cefoperazone/sulbactam is not evidence-based, and the WHO does not recommend its use in clinical practice. FDCs represent a combination of two or more active substances in a fixed ratio of doses [8]. Cefoperazone-Sulbactam is the only antibiotic FDC belonging to the *not recommended* AWaRe category currently in use in Romania.

We aim to describe the evolution of cefoperazone-sulbactam consumption in Romania between 2011 and 2024, raising awareness about the risk of antimicrobial resistance intensification and the incompatibility of cefoperazone-sulbactam prescribing with antibiotic stewardship programmes, as this fixed-dose combination is not recommended by the World Health Organisation in clinical practice as it may result in selection of antimicrobial resistance.

## Methods

We conducted a retrospective study to assess the consumption of cefoperazone-sulbactam (J01DD62) at the hospital and community levels between 2011 and 2024. The use of anonymized data on antibiotic consumption in Romania collected by the National Center for Surveillance and Control of Communicable Diseases was carried out with the approval of the National Institute of Public Health (document number 10392/04.06.2025).

The estimation of the national consumption of cefoperazone-sulbactam (J01DD62) was based on data provided by the National Health Insurance Fund and the IQVIA–Multinational Integrated Data Analysis System (IQVIA-MIDAS). IQVIA-MIDAS, formerly IMS Health, provided a commercial database containing data on pharmacy sales across the entire supply chain, including the total volume of antibiotics sold to wholesalers and hospital pharmacies by distributors. Antibiotic sales expressed in standard units (SUs) were converted into defined daily doses (DDDs) per 1,000 inhabitants per day according to the WHO Collaborating Centre for Drug Statistics Methodology [9]. The sales and reimbursement data were expressed in standard units corresponding to 1 g cefoperazone. Four SUs were converted to 1 DDD and the WHO recommended formula for obtaining the DDD per 1,000 inhabitants per day (total DDD x 1000 / population x 365) was used.

Since 2019, the National Center for Surveillance and Control of Communicable Diseases of Romania has reported differentiated antimicrobial consumption (AMC) data for the community and hospital sectors.

Data regarding the permanent resident population of Romania published by Eurostat were used for calculating the “defined daily doses (DDD) per 1000 inhabitants per day” indicator at the country level, whereas data published by the National Institute for Statistics were used at the county level. The Nomenclature of Territorial Units for Statistics (NUTS) developed by the European Union was used to calculate cefoperazone-sulbactam consumption at the macroregional level (NUTS1 corresponding to 4 macroregions) and at the county level (NUTS3 corresponding to 41 counties and the capital city) in Romania.

Data processing, handling, and aggregation were performed in Python (version 3.12.3) using the pandas (version 2.3.3) and NumPy (version 1.26.4) libraries. Temporal analysis of national consumption trends was conducted via segmented linear regression using the Ordinary Least Squares (OLS) model from the statsmodels library (version 0.14.1). Candidate breakpoints were evaluated using the Akaike Information Criterion (AIC), with lower AIC values indicating the preferred breakpoint model among those tested. Regional differences in mean county-level cefoperazone-sulbactam consumption, expressed as DDD per 1,000 inhabitants per day, were assessed using one-way ANOVA after grouping counties into the four Romanian macroregions. The Shapiro-Wilk test was used to evaluate whether the within-group distributions were approximately normal, and Levene’s test was used to assess whether variances were comparable across macroregions; both tests were implemented using SciPy (version 1.12.0). Classical one-way ANOVA was used to compare macroregional means and Tukey’s honestly significant difference test was used for pairwise post-hoc comparisons. Statistical significance was defined as a p-value < 0.05.

## Results

**Fig. 1:**
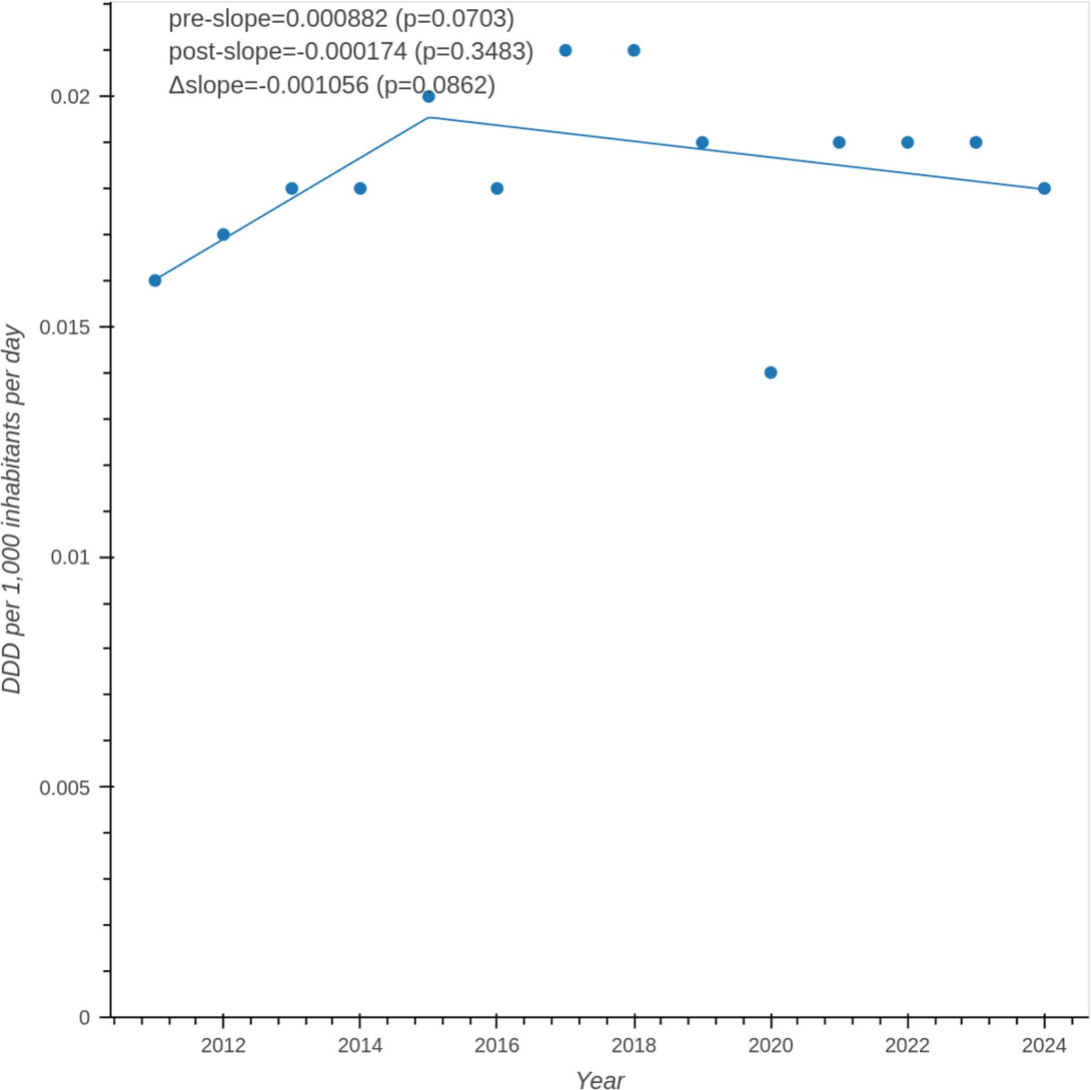
Evolution of cefoperazone-sulbactam consumption in Romania between 2011 and 2024

The consumption of cefoperazone-sulbactam in Romania between 2011 and 2024 ranged from 0.016 to 0.021 DDD per 1000 inhabitants per day, with an average of 0.019 DDD per 1000 inhabitants per day. An increase (31.3%) in cefoperazone-sulbactam consumption was observed from 2011 to 2018, followed by a decrease in the following years, with the lowest value recorded in 2020. Evaluation of candidate breakpoints using the AIC identified 2015 as the optimal breakpoint. While the model indicated a shift from an increasing trend to a decreasing trend after 2015, this change in antibiotic consumption per year was not statistically significant (*p* = 0.3). The consumption of cefoperazone-sulbactam in Romania between 2011 and 2024 is shown in Fig. 1.

The percentage of cefoperazone-sulbactam prescribed in the hospital sector between 2019 and 2024 ranged from 96.5% to 98.7%. An increase in the percentage of cefoperazone-sulbactam consumed in the hospital sector was observed from 2019 to 2024, with the highest recorded value reported in 2024.

The RO1 macroregion in the north-west of Romania registered the highest consumption, with the average county consuming 4,672 DDDs between 2021 and 2024. The proportion of cefoperazone-sulbactam consumed yearly in the same period by the RO1 macroregion varied between 40 and 53% out of the total reported at the national level.

The RO2 macroregion in the north-east of Romania registered the lowest consumption, with the average county consuming 1,392 DDDs between 2021 and 2024. The proportion of cefoperazone-sulbactam consumed yearly in the same period by the RO2 macroregion varied between 10 and 11% out of the total reported at the national level.

**Fig. 2:**
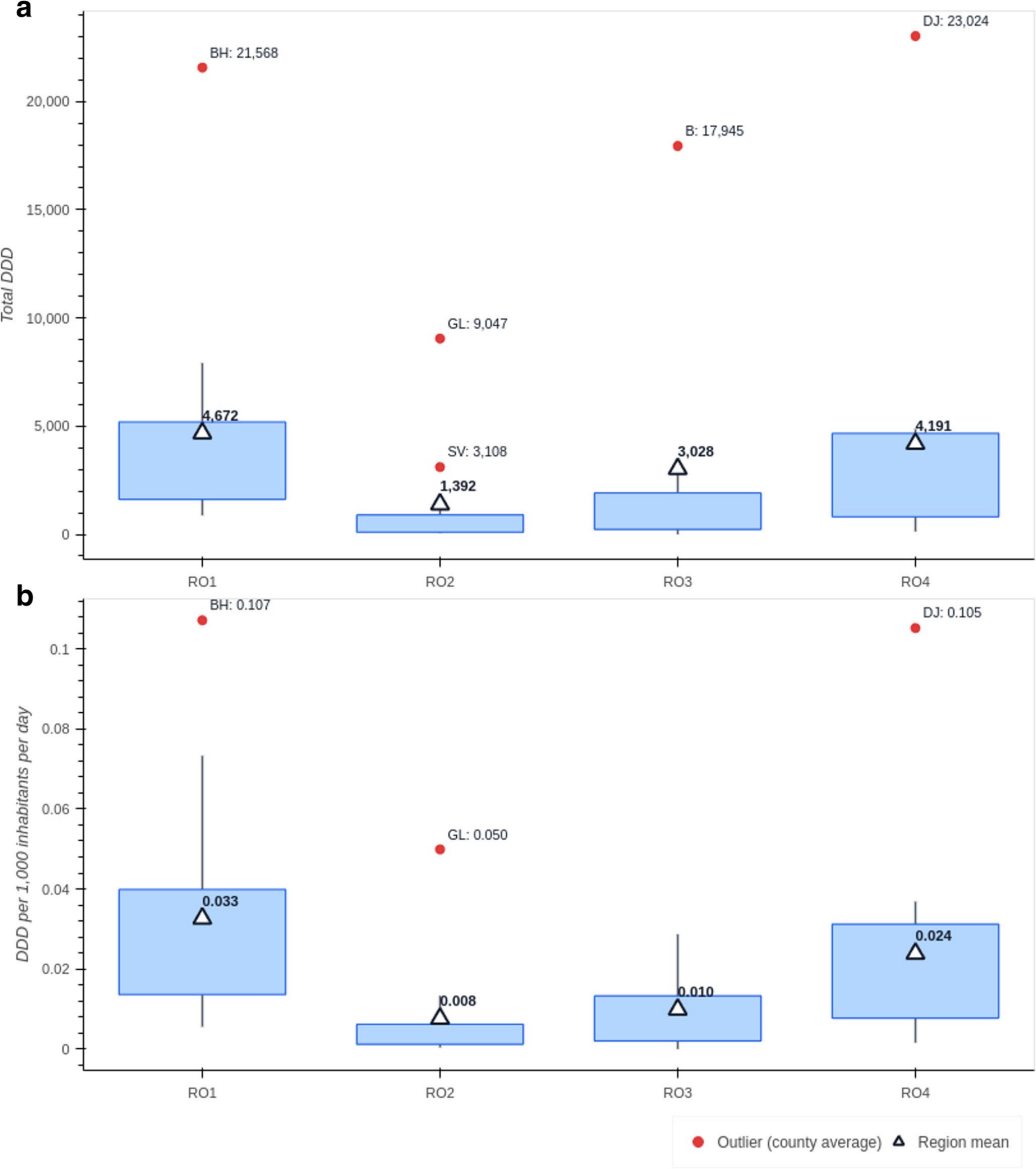
**a** Average yearly county consumption of cefoperazone-sulbactam (DDD) per macroregion between 2021 and 2024; **b** Average yearly county consumption of cefoperazone-sulbactam (DDD per 1,000 inhabitants per day) per macroregion between 2021 and 2024

The average yearly county consumption of cefoperazone-sulbactam per macroregion between 2021 and 2024 is shown in Fig. 2.

**Fig. 3:**
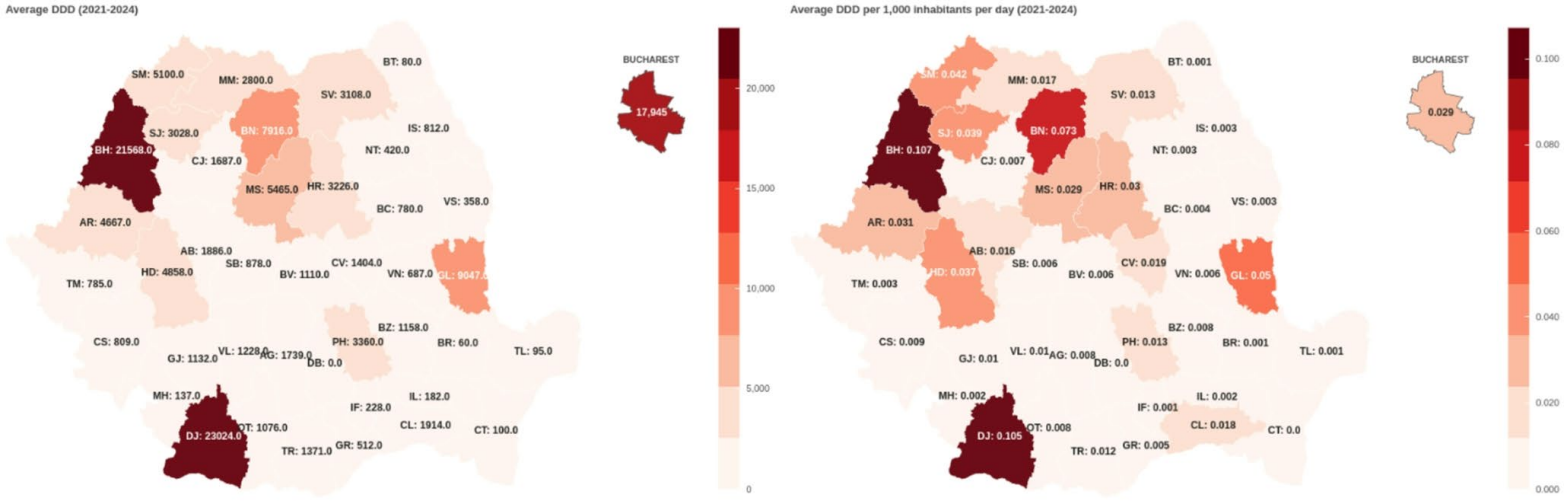
Average cefoperazone-sulbactam consumption per county between 2021 and 2024

When adjusted for population, the RO1 macroregion in the north-west of Romania registered the highest average consumption of cefoperazone-sulbactam between 2021 and 2024 (0.033 DDDs per 1000 inhabitants per day), while the lowest consumption was reported in the RO2 macroregion in the north-east (0.008 DDDs per 1000 inhabitants per day). The average consumption adjusted for population per county between 2021 and 2024 is shown in Fig. 3.

The average consumption (DDD per 1000 inhabitants per day) per county from 2021 to 2024 varied between 0 and 0.287, with an average of 0.025 (SD = 0.047). One county registered no cefoperazone-sulbactam consumption during the four-year period. The highest average consumption was registered in one of the counties in the south-west macroregion of Romania (RO4), with 0,287 (SD = 0) DDD per 1000 inhabitants per day.

**Fig. 4:**
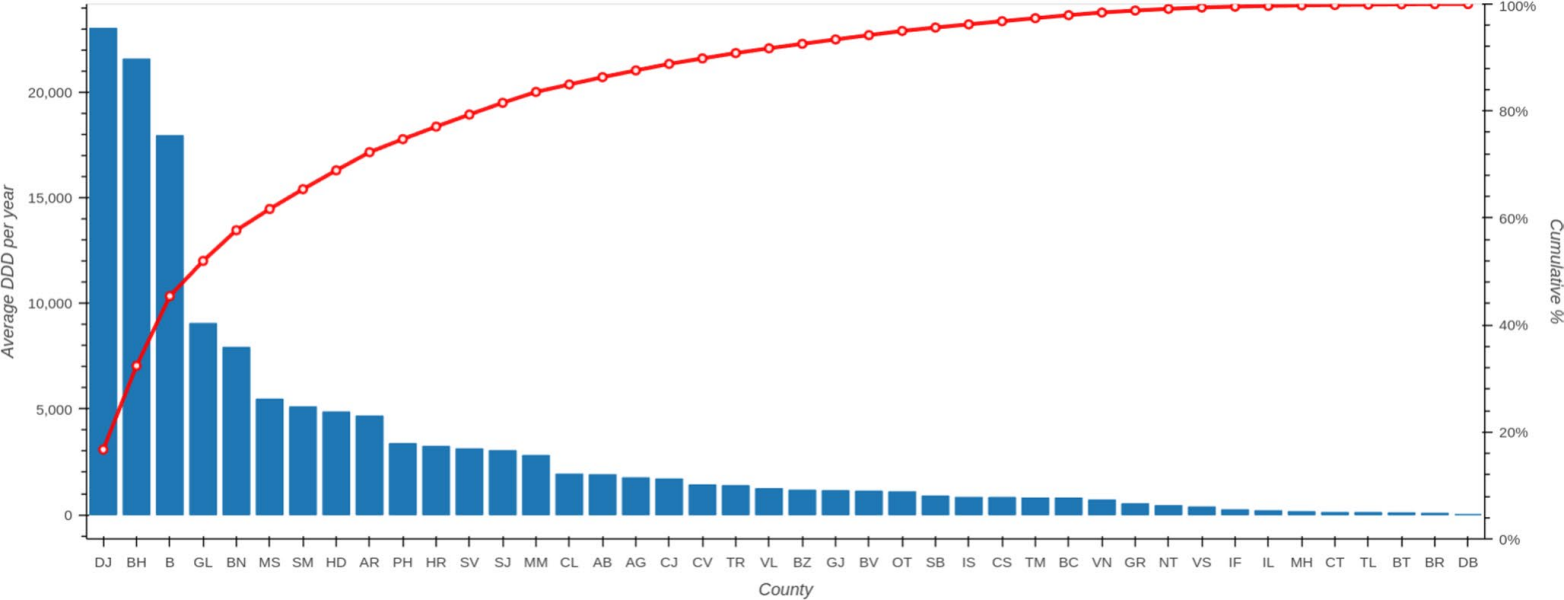
Average yearly consumption (DDD) of cefoperazone-sulbactam per county 2021-2024

The four highest consumer counties account for 47.6–55.7% of the total cefoperazone-sulbactam consumption between 2021 and 2024 (Fig. 4).

One-way ANOVA on DDD per 1000 inhabitants per day data suggested that there were no statistically significant differences between macroregions (F = 2.78, *p* = 0.054). The results of the Tukey simultaneous tests for differences are shown in Table 1. Although differences are observed in the mean values, they can be explained by counties with abnormally high consumption.

**Table 1:** Tukey simultaneous tests for differences between average cefoperazone-sulbactam consumption in DDDs per 1000 inhabitants per day per macroregion between 2021 and 2024

Region 1	Region 2	Differences of means +/- SDs	95% CI	*p* value
RO1	RO2	-0.025 +/- 0.01	(-0.051, 0.01)	0.07
RO3	R01	-0.023 +/- 0.01	(-0.051, 0.005)	0.15
RO4	RO1	-0.009 +/- 0.01	(-0.037, 0.019)	0.84
RO3	RO2	0.002 +/- 0.1	(-0.026, 0.03)	0.1
RO4	RO2	0.016 +/- 0.01	(-0.012, 0.044)	0.43
RO4	RO3	0.014 +/- 0.01	(-0.016, 0.04)	0.59

## Discussion

The decrease in the consumption of cefoperazone-sulbactam during 2016 reflects the overall decrease in the consumption of antibacterials at the national level [10]. The ESAC-Net annual epidemiological report for 2016 showed a decrease of 11.4% in the consumption of antibacterials for systemic use expressed as DDD per 1,000 inhabitants per day compared with the previous year. This decrease in antibiotic consumption can be partly explained by the implementation of new legislation that mandated the hiring of infectious diseases doctors in the Infection Prevention and Control Departments of hospitals and their responsibilities [11]. The reduction in cefoperazone-sulbactam consumption in 2020 can be attributed to the disruption of the healthcare system caused by the COVID-19 pandemic and the dominant consumption of this FDC in the hospital sector.

According to reports published by the European Surveillance of Antimicrobial Consumption Network (ESAC-Net) during the period 2010–2019, a statistically significant decrease in the total consumption of antibacterials for systemic use for the EU/EEA was observed overall [12]. This statistically significant decreasing trend continued between 2020 and 2022, as reported by the ESAC-Net annual epidemiological report [13]. Romania constantly registered one of the highest total antibiotic consumption levels in the EU/EEA between 2011 and 2023 (range = 24.4–28 DDD per 1,000 inhabitants per day). No statistically significant trend was detected for the national consumption of antibiotics between 2014 and 2023. Similarly, the consumption of cefoperazone-sulbactam at the national level has been constant, with no significant trend identified even though decreases in consumption can be observed in 2016 and 2020.

Bortone et al. reported that most countries (70,7%) sell at least one FDC not approved by the U.S. Food and Drug Administration (FDA) and that cefoperazone-sulbactam is available for sale in 28 countries worldwide, ranking 4th in the list of FDCs available in most countries. It was estimated that cefoperazone-sulbactam was the 10th most sold FDC in 2015, with 0.16 × 109 standard units, based on the IQVIA pharmaceutical database [14]. According to the same study, Romania registered the 5th highest percentage of FDCs not approved by the U.S. FDA out of the total antibiotic sales among EU countries.

The parenteral route of administration and the lack of a legislative framework for outpatient parenteral antimicrobial therapy in Romania explain the predominant consumption of cefoperazone-sulbactam in the hospital sector. Regardless, a small percentage (1–3%) of the total quantity of sulbactam-cefoperazone consumed in Romania is sold in community pharmacies every year. A legislative framework for the regulation of antibiotics dispensed through community pharmacies has been in place since 2024 [11].

The target set by the WHO of at least 60% of total antibiotic consumption being *Access* group antibiotics introduced in 2019 represented a challenge for Romania, who reported values between 49 and 52.8% for this secondary outcome indicator between 2019 and 2023. The percentage of *Access* group antibiotics consumption decreased from 52.8% in 2019 to 51.2% in 2023. The EU Council Recommendation 220/2023 set the target of 65% *Access* group consumption out of total antibiotics for 2030 for all member states [15]. In line with both the WHO recommendations and the EU Council recommendations, the following measures were proposed to the Romanian Ministry of Health in April 2025:

1. Training activities to increase awareness of the risks associated with cefoperazone–sulbactam use, with a particular focus on counties with high consumption of this antibiotic.
2. Restricting hospital use of cefoperazone-sulbactam through a special authorization form dependent on approval from an infectious disease specialist).
3. Elimination of the possibility for cefoperazone-sulbactam to be dispensed through community pharmacies.
4. Implementing discouraging measures for the import of cefoperazone-sulbactam.

Corrective initiatives should be tailored to our country profile. According to Hofstede’s model of cultural dimensions, Romania has a high rating (90/100) in uncertainty avoidance (UAI) [16]. Doctors from high-UAI countries reported less than optimal perceptions of guidelines and did not express enthusiasm for multidisciplinary involvement [17]. This cultural driver can explain why the consumption of cefoperazone-sulbactam has remained stable at the national level despite the high percentage of hospitals (89%) having guidelines for antimicrobial prescribing according to the infection prevention and control assessment framework report published in 2025 [18].

The rejection of the reclassification proposal of cefoperazone-sulbactam in the updated 2025 WHO AWaRe classification of antibiotics calls for the discontinuation of cefoperazone-sulbactam use in Romania. While educational and training activities focused on the outlier counties are necessary to raise awareness about the risk of increased selection of antimicrobial resistance exerted by cefoperazone-sulbactam on *Acinetobacter baumannii*, “the ultimate responsibility for the policies to ensure the prudent use of antimicrobials lies with the national, regional and local governments” according to the guidelines for the prudent use of antimicrobials in human health [19].

## Limitations

The analysis was limited by the quality of the data provided by the National Health Insurance Fund and the IQVIA–Multinational Integrated Data Analysis System (IQVIA-MIDAS). The major limitations of reimbursement data provided by the National Health Insurance Fund is that it excludes antimicrobials dispensed without a prescription and prescribed antimicrobials for which reimbursement data was not claimed. Thus, reported sales for the community sector were used in order to provide a more accurate estimate of the total consumption at the national level since this data includes consumption without prescription and non-reimbursed.

A further limitation of this study is the absence of data regarding the specific clinical indications for the cefoperazone-sulbactam prescription, as no data is collected regarding the diagnosis, aetiology of the infection treated or the medical specialty of the prescribing physicians.

## Data Availability

The datasets used and/or analyzed during the current study are available from the corresponding author upon reasonable request.
